# Mental health and psychosocial support in conflict: children’s protection concerns and intervention outcomes in Syria

**DOI:** 10.1186/s13031-021-00350-z

**Published:** 2021-04-01

**Authors:** Nada Raslan, Arran Hamlet, Veena Kumari

**Affiliations:** 1 Division of Psychology, Department of Life Sciences, College of Health, Medicine and Life Sciences, Brunel University London, Kingston Lane, Uxbridge, London UB8 3PH UK; 2 Department of Infectious Disease Epidemiology, MRC Centre for Global Infectious Disease Analysis, Imperial College London, London, UK; 3 Centre for Cognitive Neuroscience, College of Health, Medicine and Life Sciences, Brunel University London, UB8 3PH Uxbridge, UK

**Keywords:** Syria, Mental health and psychosocial support, Toxic stress, Child protection, Psychosocial deprivation, Depression, Anxiety, Displacement, Conflict

## Abstract

Child protection and mental health during conflict intersects with a variety of adverse conflict-related factors, and intervention outcomes in the field are often difficult to predict. Using the casefiles of 376 school children registered in a Mental Health and Psychosocial Support (MHPSS) project in the Northwest governorate of Idleb in Syria, this study aimed to determine (i) the rates of various protection concerns (potential mental health conditions, psychosocial deprivation issues, and social, behavioural and emotional issues) for students enrolled in this project, (ii) whether the rates of any of the protection concerns varied between children and adolescents, or between boys and girls, and (iii) which of the identified demographic and protection sector factors predicted the presence of potential mental health conditions and MHPSS intervention outcomes. MHPSS interventions (including individual MHPSS sessions tailored for children in conflict, resilience building activities, tutoring, peer building activities, community awareness, and other tailored services) were implemented at schools operated by the UK-based organization, Syria Relief. The variables tested included demographic variables of age group (208 children, aged 4–9 years; 168 adolescents, aged 10–14 years) and gender (211 males, 165 females), and 23 protection sector variables including 11 potential mental health problems (anxiety, attention deficit hyperactivity disorder, conduct disorder, autism, epilepsy, motor tics, depression, post-traumatic-stress disorder, social phobia, specific phobia, learning disability), 7 psychosocial deprivation (PSD) variables (war injury, child labour, loss of caregiver, neglect, domestic abuse, displacement, poverty), and 5 social, behavioural and emotional (SBE) variables (low/abnormal socialization, emotional issue, peer issues/being bullied, peer issues/being aggressive, educational decline). Within the sample, 73.7% were found with a probable mental health problem, with 30.6% showing signs of anxiety, 36.2% of depression and 26.6% showing signs of post-traumatic-stress disorder. Additionally, 74.5% of the sample had at least one form of PSD present (42.6% were displaced, 39.6% suffered from abject poverty), and 64.9% had a reported SBE concern. Children were more likely to have a potential mental health concern, especially autism and PTSD, and poor socialization; while adolescents were more likely to engage in child labour, experience abject poverty, exhibit aggressive behaviour, and educational decline. Male gender was associated with child labour and aggressive behaviour while female gender was associated with the presence of potential mental health problems, especially depression, and loss of caregiver, and poor socialisation. Odds ratios (ORs) indicated significant negative impact of the presence of SBE concerns (any), 4.45 (95% CI: 1.68–12.7), emotional issue, 11.02 (95% CI: 2.76–74.49), low/abnormal socialization, 8.37 (95% CI, 2–57.71), and displacement, 2.91 (95% CI, 1.21–7.48) on the child’s mental health. MHPSS intervention outcomes were categorized as case improvement, decline, or incomplete/limited information available; with case improvement noted for 63.6% of the sample, decline noted for 14.4%, and incomplete treatment/limited follow-up noted for 22.1% of the sample. Additional analysis of predictors of treatment success found that child labour was significantly associated with a lack of treatment success, OR 0.24 (95% CI, 0.07–0.92). These findings provide important insights into the complex tailoring needs that protection and MHPSS field projects require.

## Background

In nearing its tenth year, the Syrian conflict has claimed the lives of 200,000–500,000 civilians including at least 29,000 children, and destroyed the majority of the country’s services and infrastructure. Over 11.7 million people require humanitarian support, including 5 million children below the age of 18 [1]. Various grave violations of human rights have taken place, and the complexity of mental health and protection needs have been further compounded by the increase of abject poverty, gender-based violence (GBV), child exploitation, child marriage, child labour, exposure to physical events of violence and siege situations, shelling, repeated displacement, and loss of family and social resilience systems [1]. Prior to 2011, Syria was a country that had an 86% literacy rate and possessed extensive social capital, while at this current time 6 million civilians have fled the country and over 7 million are internally displaced; meaning over 50% of the pre-war population has forcefully relocated [2, 3]. Globally, Syria has the highest rate of Internally Displaced Persons (IDPs), and these demographic shifts have led to a drastic decline in various essential services including education and healthcare. Since 2011, over 40% of all schools, hospitals and infrastructure have been destroyed or rendered out of use; at a time where healthcare is most needed, and education stands as one of the primary preventative barriers for nearly all forms of child exploitation [3, 4]. This has been further exacerbated by systematic campaigns of censure and violence against education, healthcare, and humanitarian workers. For the majority of the conflict, Non-Governmental Organizations (NGOs) provided services to the most at-risk civilians across the country – ranging from projects in education, healthcare, mental health and psychosocial support (MHPSS), infrastructure reservicing, livelihood, emergency food and shelter support, to various other services based on local needs [1, 4–6]. Despite this, aid funding and support continues to decrease; in 2019 only 65% of funding appeal requirements were met in Syria, a decrease of 7.4% from 2018 [1, 6].

The Northwest of Syria has increasingly become a focal point for both humanitarian need and cross-border aid activities, housing the vast majority of IDPs in the governorates of Idleb and Aleppo throughout the duration of the conflict [7]. High levels of competition for livelihood opportunities and essential services further exasperates poverty and needs. Vast numbers of makeshift IDP camps are now scattered over the agricultural northern landscape of Syria, often cut off from essential services and rendering these communities extremely vulnerable and in need of support [1, 7]. In 2019, a variety of highly aggressive military offensives began taking place in the North of the country where the highest concentration of civilians remains in need of support. Conditions in the North of the country are constantly shifting, dire, and remain largely obscure to those outside of Syria [1, 7].

### Protection and psychosocial deprivation concerns in Syria

Psychosocial deprivation (PSD) concerns; referring to extremely negative social and intellectual elements of an environment – are virtually synonymous to conflict and instability, and over 97% of communities in Syria reported having at least one substantial PSD or protection related threat in 2018 [5, 8]. Syria’s protection needs in the Humanitarian Needs Overview Report (HNO) (2019) looked extensively into all protection needs for Syria in 2018, despite physical limitations and lack of authorization which prevents the collection of protection-specific information [1]. Of the protection and PSD concerns specific to children, child labour was found to be a leading concern (reported in 80% of the communities screened in the report), followed by child marriage (45%) [1]. Earlier child-protection data from the HNO 2017 report points to concerns including family separation in 52% of the communities screened, followed by domestic violence (51%), child recruitment (47%), explosive hazards (42%), and sexual violence (27%) [5]. Such concerns place all segments of the population in a state of prolonged and extreme stress, which if left unaddressed can have negative social outcomes for years to come [9–11]. Negative socioeconomic effects of war such as unemployment, poverty, and loss of housing and communal networks are present in the majority of Syrian communities both within and on the outskirts of the country, as various needs assessments from humanitarian organizations concluded; these are often found to directly contribute to more extreme forms of psychosocial deprivation and extreme prolonged stress (‘toxic stress’) for the entire population [1, 12–14].

Childhood toxic stress in particular is a long-term state that be differentiated from normal levels of stress or from a singular traumatic event – wherein the child experiences prolonged exposure to adversity, including any form of emotional and physical abuse, neglect, chronic caregiver distress or illness, prolonged exposure to violence, the absence of reliable adult support systems when needed, and general instability generated through conflict [14, 15]. The UN estimates that one in three school children are displaced in Syria, placing nearly one third of Syrian children in this state of toxic stress [7, 14]. As a result, the effects on their mental health is not simply the result of a singular isolated event – but that of their constantly shifting and highly unstable environment [16]. This differentiation is important for project implementation and research, as it speaks to the need for broad and intersectoral approaches in children’s MHPSS projects, and for the integration of protection standards across various sectors and types of projects [17, 18].

### Mental health and psychosocial support in Syria

Corresponding with the physical damage, the overall impact on mental health of the Syrian population remains understudied and highly underestimated in its severity of consequence – despite this conflict resulting in one of the largest humanitarian crises since World War II in terms of displacement, infrastructure damage and violence rates [1]. Prolonged exposure to such conflict-related PSDs for both children and adults can lead to long-term cycles of psychosocial distress and violence which persist for years post-conflict [15, 19–21]. Studies have linked such prolonged trauma to post-traumatic stress disorder (PTSD), depression, anxiety, drug abuse, heart disease, diabetes, and other stress related illnesses, and such negative outcomes can result in cycles of negative socioeconomic and cultural development in the long-term [22]. Mitigating interventions such as education, protection, and MHPSS activities can support community resilience and development both proactively and reactively, and may provide tools to lessen the long-term negative effects of conflict [21, 23, 24]. These projects are often unique in their need to be integrated within other sectors, and their more sensitive nature warrants extensive project tailoring that has to consider local community practices and values; as such, these types of projects can highly benefit from more research support at the project level.

Physical and mental health research has often found that prolonged exposure to severe trauma, stress, and uncertainty (‘toxic stress’) especially during childhood and adolescence, can be associated with chronic physical illness including heart disease, a variety of cancers, stroke, and more severe mental health disorders such as schizophrenia and dysthymia later in life [8, 10, 25–28]. Children and adolescents are particularly vulnerable to the long-term negative effects of toxic stress, which can disrupt their developing neuro-endocrine-immune response through prolonged stress and cortisol activation [9, 15, 17]. Ultimately this keeps the immune and neurological systems in a constantly inflamed state, with the inability to ‘switch-off’ – disrupting normal physiological development in some cases [9, 15, 29]. Without adequate care and resilience systems in place, this can lead to cognitive impairment and severe disruption to their normal brain development, as well as disruptions to all stress-related organ systems which places them at a higher risk for a variety of chronic physical and mental illnesses persistent in adult life [29–32].

Both the determinants and impacts of mental health can be linked to the greater physical environment, and as such a multi-faceted approach is often implemented when addressing these concerns in a conflict setting [32]. The World Health Organization (WHO) estimates that the prevalence of mental health disorders is higher in fragile and conflict settings (FCAS), with approximately 1 in 5 people living FCAS suffering symptoms of depression, PTSD, anxiety, bi-polar disorder, or schizophrenia [33]. Social determinants of mental health continue to gain more focus in recent years, with an increased understanding in how demographic, economic, environmental, and cultural conditions can impact mental health – beyond biological and developmental explanations [21, 22, 32, 34].

Similarly, unaddressed mental health concerns can significantly impact communal socioeconomic development. The World Economic Forum estimates that over half of the economic burden of disease will be accounted for by mental health illness – adding up to more losses than that of cancer, diabetes and chronic respiratory illnesses combined [23, 24, 35]. A variety of interdisciplinary fields ranging from global healthcare to economics are gaining more insight to the larger consequences of poor mental health, and the costs this can have on the sustainable development of a given population [11, 19, 26, 36]. In recognizing these associations between mental health and the environment, addressing mental health in FCAS is often exceptionally complex and requires a high degree of local service provider coordination, cross-sectoral project tailoring at community levels, and a significant amount of accountability and monitoring throughout. It’s important to be sensitive and aware of local cultures, practices, and experiences; as mental health interventions can be stigmatizing or unintentionally harmful without adequate community buy-in [18]. MHPSS intervention success is often linked to activities that can be sustained long-term, and these are often integrated within already-established community structures such as schools, hospitals, community and religious centres, local healing practitioners, and established social networks [18]. Additionally, psychiatric care in conflict settings is often very difficult to implement or sustain on an individual basis, and therefore a wide variety of MHPSS activities are usually implemented to fill the gap [18]. These activities can range from advocacy campaigns, group trust building activities, community awareness projects, tailored activities through schools, child or female friendly space building, story-telling exercises, Psychological First Aid (PFA), MHPSS service mapping and information sharing, and various multi-sectoral interventions tailored for the conditions or beneficiary group in question [18, 37]. The wide range of MHPSS and protection activities are bound by a similar objective; in that they attempt to actively support and prevent mental health and protection issues from worsening for the most vulnerable civilians in FCAS [18, 37–39].

### The present study

The main aims of this study are to empirically determine the most prevalent protection concerns in a single student sample, as well as identify the protection and demographic factors that might predict the presence of potential mental health issues, and the outcomes of the MHPSS project. This is based on a detailed retrospective analysis of a comprehensive dataset generated in the context of a single education-centred MHPSS protection project in the Northwest of Syria implemented during the conflict.

Specifically, we firstly examine the prevalence rates of various protection concerns; namely potential mental health issues (anxiety, attention deficit hyperactivity disorder, conduct disorder, autism, epilepsy, motor tics, depression, post-traumaticstress disorder, social phobia, specific phobia, learning disability), PSD concerns (war injury, child labour, loss of caregiver, neglect, domestic abuse, displacement, poverty), and social, behavioural and emotional (SBE) functioning concerns (low socialization, emotional issues, peer issues, and educational decline) for 376 students who were enrolled in a MHPSS protection project in the Northwest governorate Idleb in Syria. The second aim was to examine whether the prevalence rates of these specific protection concerns varied demographically between younger (aged 4–9 years) and older (10–14 years) children, or between male and female children in a cross-sectional assessment. Our third and final aim was to examine which of these demographic and protection variables may predict the presence of potential mental health concerns and MHPSS intervention outcomes using predictive logistic regression analyses.

## Methods

The data were obtained directly from Syria Relief – the largest Syria-focused, UK-based NGO. Approval for the use of these data for research purposes was given by Syria Relief prior to the data being shared with the researchers; and the use of these data for the present study was approved by the College of Health and Life Sciences Research Ethics Committee at Brunel University London (Reference: 17634-LR-Jul/2019–20,011-1).

Syria Relief has maintained largescale access across the country with ongoing operations in the Northwest of Syria since 2011. To date, one of their largest areas of experience is in the education sector – with the program having already supported over 20,000 students (including 2,000 children with special needs). Syria Relief currently runs over 300 schools with at least one mental health professional at every school [13]. In tandem with their education projects, and as part of their protection specific projects, Syria Relief operated over 15 Child Friendly Spaces (CFS) in 2019. These spaces act as resilience-building and risk-mitigating spaces in response to the toxic stress that children in Syria are regularly exposed to. CFS’ also provide a site for more focused MHPSS activities with trained psychologists, counsellors, case managers, social workers, and other caregivers; ensuring group and individual support to students as needed. More than 13,750 children frequented these CFS' between 2011 and 2019, and continue receive MHPSS on a regular basis through Syria Relief programs [13].

The utilization of schools for MHPSS intervention serves as an effective entry point into the community and children in question. Syria Relief’s implementation activities include a variety of planned actions guided by various best practice tools including those compiled by the Global Protection Cluster (GPC), the Inter-Agency Standing Committee (IASC) Guideline’s for Emergency Settings, the Minimum Standards for Child Protection in Humanitarian Action (CPMS), and various others [18, 37–39]. MHPSS and protection interventions largely require multi-faceted forms of support, which can be generally categorized into four ‘tiers’ or layers of support as seen in the IASC’s intervention pyramid [18].

The four-tier actions, as carried out by Syria Relief, firstly prioritize the provision of basic needs and security to the highest risk children and their families; often done through provision of clothing, financial voucher distributions, food and hygiene packages, and referrals in cases of homelessness or similar situations where a child lacks the most basic needs of food, shelter, and security.

The second tier of the IASC protection pyramid and Syria Relief’s interventions include community engagement through psychosocial support (PSS) activities [18]. For the project that provided data for the current study, such activities included awareness campaigns on subjects such as child labour, marriage and recruitment, child wellbeing, on the importance of MHPSS including warning signs and identification of at-risk children, and distributing information about local MHPSS services. This also included referral and support for children who are orphaned, separated from their families, or in need of additional community-based care and support.

The third layer then focuses on non-specialized support activities, often implemented directly at the schools. Children enrolled in the Syria Relief MHPSS program undergo more focused, non-specialized support such as direct family interventions in the cases of child labour and suspected PSD concerns at home. CFS' in the schools are regularly utilized to allow for safe spaces where various guided art, sport, play, peer interaction, and skill-building activities can be practiced. These – activities are locally tailored at community level to develop skills around mindfulness, self-reliance, cooperation, resilience building, and empowerment; approved by the local education directorate of Idleb for service coordination and buy-in, as well as ensuring local norms and values are respected at all stages [13, 18]. The majority of cases in this sample receive support up to this level by trained and supervised MHPSS caseworkers.

The final layer of the IASC MHPSS pyramid includes specialized focused services and treatment [18]. While Syria Relief did not provide such specialized psychological treatment through this particular project, the organization has access to psychological staff in Idleb and extensive knowledge of services in the area, as well as an established network and referral mechanism in place where required. This referral system includes coordination with mental and physical health service providers in Idleb – despite their limited numbers. Local coordination between service providers helps to maintain best practice knowledge sharing, as well as aid case managers in tracking the progress of higher risk cases [37]. Syria Relief employs psychologists and/or trained mental health staff as part of their protection team and at every school; offering trainings to teachers, parents, and MHPSS staff, as well as providing some specialized treatment and supporting MHPSS activity tailoring [13].

### Participants

The sample consisted of 376 children and adolescents that were 4–14 years old (211 males, 165 females; M = 8.99, SD = 2.25 years of age) from three primary and secondary schools in three rural towns in the Idleb governorate, located in the Northwest of Syria. All registered students are screened for protection related concerns by trained education, mental health, and/or protection staff in Syria Relief’s protection and education programs. Students were registered in the MHPSS project due to various health, PSD, or SBE concerns between the years of 2017 and 2019; the causes for registration in the MHPSS program guided the selection of protection variables to be examined in this study.

All three schools are registered with the local education authorities of Idleb to ensure community buy-in, quality assurance, and coordination between other education and MHPSS providers in the region. All teachers, case managers and protection staff working for Syria Relief are trained to screen students for protection related concerns, using the Global Protection Cluster (GPC) and Protection Cluster Coordination (PCC) trainings; and they have support from mental health professionals at every school. As part of Syria Relief’s minimum best practice standards and capacity building policies, regular staff training is carried out to support more streamlined project implementation and information gathering across projects and offices; and to accurately measure protection project progress. As a result, identifying children’s concerns, measurement indicators, case progress and reporting practices are fairly standardized.

The data were aggregated for the purpose of case management and Monitoring & Evaluation (M&E) activities by the Syria Relief protection team. The data included location, age, living status (from the host community or internally displaced), gender, level and type of risk on the child, date registered for MHPSS, and the number of PSS sessions that took place. The case manager wrote case descriptions, comments, and details of the progress of each case prior to, throughout, and after the planned MHPSS activities concluded. Prior to the school-year commencing, all education and protection staff are ensured up-to-date trainings which includes best practices around case-management and M&E in protection and education in Syria. This information was collected over a three-year period (2017–2019), and regularly tracked in a single Microsoft Excel file by the Syria Relief protection team who use this to assess against the project’s expected outcomes and progress guided by globally recognized guidelines and standards for child protection in humanitarian settings, including those by the GPC, IASC, WHO, Sphere Standards, and CPMS, among other protection and education centred criteria [18, 38, 39]. Casefiles also indicated either case improvement, decline, or incompleteness due to absence or lack of participation and data.

Of the 376 children, 160 were classified as IDPs with the remaining from the host community. In this the sample, 204 children were from the ages of 4–9 and 172 adolescent children were aged 10–14 years; 211 of the students were male and 165 were female.

### Mental health assessment tools

The data categorizations and assessment of the cases was carried out retrospectively by the researchers, using casefile data and using screening tools to categorize the sample further. For the screening of potential mental health disorders in the sample by the researchers, the DSM-IV referenced Child Symptoms Inventory-4 (CSI-4) and Youth Inventory-4R (YI-4R) were selected as age-appropriate symptom screening tools of potential mental health concerns using the detailed case-managers’ notes in Syria [40–43]. The CSI-4 covers symptoms of 15 common childhood mental health disorders for 5–12-year-old children.^1^ The YI-4R covers common symptoms for 12–18-year-old adolescents for 18 emotional and mental disorders [41].^2^ For this study, disorders such as separation anxiety disorder, obsessive compulsive disorder, oppositional defiant disorder, dysthymic disorder, pervasive developmental disorder, Asperger’s disorder, bipolar disorder, somatization disorder, anorexia nervosa, bulimia, and drug use, were excluded from the categorizations as the data would not allow for reliable inferences on these cases to be made. Using the screening tools and any officially noted diagnoses in the casefiles, the sample was screened and categorizations were retrospectively created to denote potential presence of symptoms of anxiety, attention deficit hyperactivity disorder (ADHD), conduct disorders, autism, epilepsy, motor tics, depression, PTSD, social phobia, specific phobias, and learning disability.

### Data extraction method

The casefiles were compiled directly to Microsoft Excel in Arabic by the Syria Relief protection team in Syria, and anonymised on-site between the years of 2017 and 2019. Consent for the use of information was obtained or denied by every child and/or their caregiver upon registration in the MHPSS project, using the standard GPC’s Arabic consent form for use of data and any other case information as Syria Relief requires, as long as any identifying information is anonymised and information sharing limited. The current dataset was limited to those with signed consent by the child and/or guardian. After acquiring consent and ethical approvals from Syria Relief and the College of Health and Life Sciences Research Ethics Committee at Brunel University London, the file was sent to the researchers for translation and coding.

All data and supplementary documents were reviewed and translated into English on a case-by-case basis by the lead researcher, who has prior experience in medical translation. The data were then coded to categorise for demographic factors (age group, gender) and qualitative data rated to denote the presence or absence of each of the identified 23 MHPSS and protection concerns for each individual case. The senior researching psychologist oversaw the screening and initial coding process, the creation of mental health categorizations, and monitored the screening process for potential presence of mental health variables, ensuring this was in line with the CSI-4 and YI-4R screening tools being used.

A total of 23 categories of protection and MHPSS concerns were created by the researchers, based on the casefile notes. These included 11 potential mental health concerns (anxiety, ADHD, conduct disorder, autism, epilepsy, motor tics, depression, PTSD, social phobia, specific phobia, and learning disability), 7 PSD variables (war injury, child labor, loss of caregiver, neglect, domestic abuse, displacement, poverty), and 5 SBE concerns (low/abnormal socialization, emotional issues, peer issues/being bullied, peer issues/being aggressive, educational decline). Physical health variables were excluded from the analysis due to limited information on these cases, as treatment was being received elsewhere.

For indicating the presence of potential mental health concerns, the researchers used the CSI-4 and YI-4R to retrospectively screen the sample for symptoms of potential mental health issues and any associated factors. These were determined on an individual case-by-case basis and reviewed by two researchers, with the senior researcher being a licensed psychologist and having experience with these tools, and in interactions between PSD and mental health [8]. The mental health variables included potential presence of anxiety, ADHD, conduct disorder, autism, epilepsy, motor tics, depression, PTSD, social phobia, specific phobia, and learning disability based on the casefile notes from the field.

For the classification of PSD concerns, the information was indicated for each case by the case managers, with notes specifying the presence of war injury, child labour, loss of caregiver, neglect, domestic violence, displacement, and poverty. Staff are trained to screen and categorize children based on these concerns and they are immediately enrolled in the MHPSS program if any are present.

The SBE categorizations denote additional social protection causes for enrolment in the MHPSS program. This included registration due to low/abnormal socialization, emotional issues or distress, peer issues (being bullied), peer issues (being aggressive), and/or educational decline. The identification of these concerns was noted by protection staff trained to specify such issues. Syria Relief staff are trained to screen for and address these SBE issues, with the casefile notes having indicated the presence and progress of these concerns. Trainings are conducted for various aspects of MHPSS and psychological assessment and support, protection in education, and protection information and data management, among various other areas to identify children with the listed SBE concerns. Educational decline was monitored by the teachers, and was noted for a number of students if they were significantly behind their age group in terms of educational attainment, or are unable to keep up with their peers in the standard classroom setting.

As for MHPSS outcomes, qualitative assessments on case improvement (positive changes), decline (negative changes) or incomplete data was indicated in the casefiles. This was categorized as case improved, declined or incomplete. Case progress notes were provided for the initial condition of the child, as well as noted changes during and after the MHPSS activities were concluded. MHPSS intervention outcomes were noted as incomplete for students that stopped attending school or where the PSS plan was not completed.

### Statistical methods

The first method of statistical analysis addressed the first two aims of this research: (i) to understand the frequency distributions of all MHPSS concerns in the sample, and (ii) to examine if and how various MHPSS and protection concerns vary by the demographic variables of age group and gender. To achieve these aims, frequency distribution tables were created to reflect the values of each of the demographic and protection/MHPSS variables in the sample. We then tested for any significant variations between the two age groups (children aged 4–9 years, adolescents aged 10–14 years) and gender (male, female) in the categorical MHPSS variables (presence or absence of potential mental health problems, PSD or SBE concerns) using Pearson’s Chi Squared Test of Independence in IBM SPSS (version 26). Every chi-squared independence test was computed on a 2 × 2 table with *p*-value significance levels differentiated at three different levels (*p* ≤ 0.05, *p* ≤ 0.01 and *p* ≤ 0.001) to account for potential Type I error inflations. Continuity corrections were applied where observed variable frequencies (*f*) were less than 5.

The final aim of this research was (iii) to examine which of the demographic and protection/MHPSS variables affected the presence of potential mental health concerns and MHPSS intervention outcomes. To achieve this, two sets of logistic regression analyses were carried out, one evaluating the association between covariates (age, gender, all PSD variables and all SBE variables) and the presence of potential mental health problems, and another evaluating the relationship between all potential mental health, PSD and SBE variables and case improvement, decline or incompleteness.

Initially, covariates were examined for collinearity through a correlation matrix and no covariate correlations were found to be over 70%. Covariates were fit to the outcome investigated (presence of a potential mental health issue or outcome of MHPSS intervention) using a hierarchical logistic regression. Here we allowed the intercept to vary between whether or not the individual was an IDP or from the host community. This allows for our analysis to account for the systematic differences in their living status (IDP or host) at outcome that may be associated with these factors. The final model was created using a systematic forward and backwards stepwise technique which pruned the covariate selection to only those that improved the model likelihood (AIC).

The predictive accuracy of these models was then evaluated using the area under the receiver operator curve (AUC); which is a measure of the sensitivity and specificity of predictions. The significance of individual covariates is shown in their *p* values and odds ratios. This analysis was carried out in R 3.4.

## Results

### Frequency distributions and association results

**Fig. 1:**
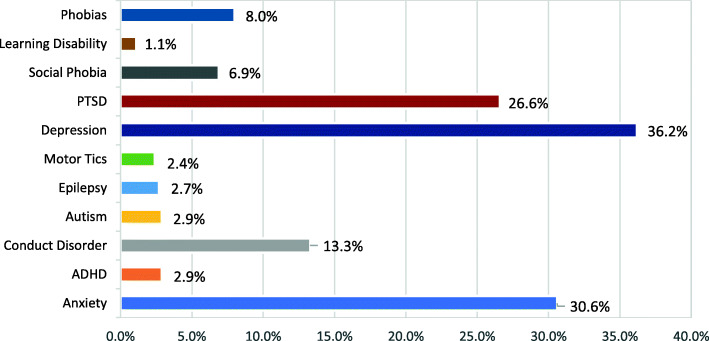
Prevalence Rates of Potential Mental Health Conditions

**Fig. 2:**
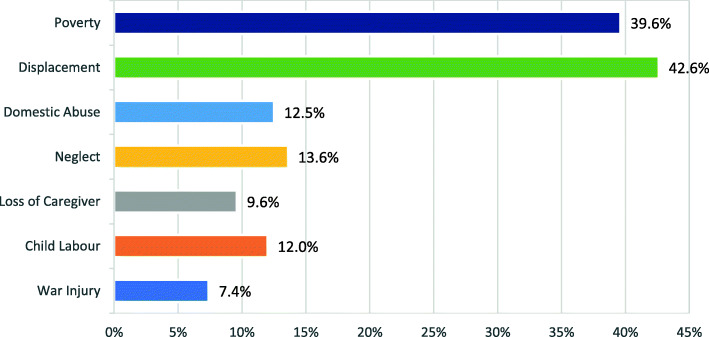
Prevalence Rates of Psychosocial Deprivation (PSD) Concerns

**Fig. 3:**
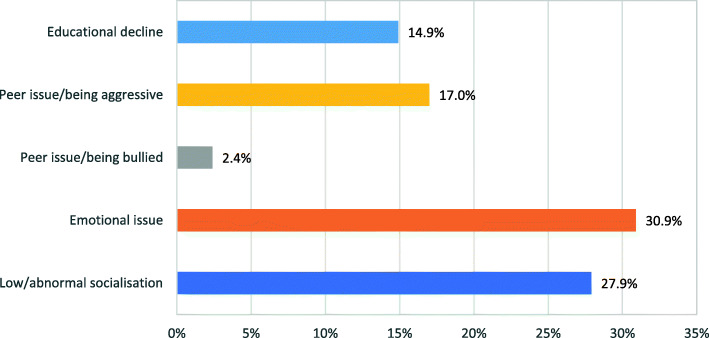
Prevalence rates of social, behavioral and emotional (SBE) concerns

In order to clearly understand the distribution and rates of different protection and MHPSS concerns, frequencies (of potential mental health problems, PSD and SBE variables) are expressed in percentages for the entire sample in Figs. 1, 2 and 3, and presented further classified by age and gender in Tables 1, 2, 3, 4, 5 and 6.

**Table 1:** Frequency distributions and Pearson’s chi-square values of potential mental health condition by age group

Potential mental health condition	Total sample (***N*** = 376)	Age group			
		4–9 Yrs. (***N*** = 208)	10–14 Yrs. (***N*** = 168)	χ^**2**^(df = 1)	***P*** value
	***f*** (%)	***f*** (%)	***f*** (%)		
Mental health condition (any)	277 (73.7%)	158 (76.0%) ↑	119 (70.8%)	1.26*	0.046
Anxiety	115 (30.6%)	62 (29.8%)	53 (31.5%)	0.13	0.716
ADHD	11 (2.9%)	6 (2.9%)	5 (3.0%)	0.00	1.000^a^
Conduct Disorder	50 (13.3%)	27 (13.0%)	23 (13.7%)	0.04	0.840
Autism	11 (2.9%)	10 (4.8%)↑	1 (0.6%)	4.42*	0.036^a^
Epilepsy	10 (2.7%)	7 (3.4%)	3 (1.8%)	0.39	0.533^a^
Motor Tics	9 (2.4%)	7 (3.4%)	2 (1.2%)	1.07	0.302^a^
Depression	136 (36.2%)	79 (38.0%)	57 (33.9%)	0.66	0.416
PTSD	100 (26.6%)	64 (30.8%)↑	36 (21.4%)	4.15*	0.042
Social Phobia	26 (6.9%)	13 (6.3%)	13 (7.7%)	0.32	0.572
Learning Disability	4 (1.1%)	3 (1.4%)	1 (0.6%)	0.84	0.771^a^
Phobias	30 (8.0%)	15 (7.2%)	15 (8.9%)	0.37	0.541

*p ≤ 0.05; **p ≤ 0.01; ***p ≤ 0.001; ↑ Higher scores; ^a^Continuity Correction applied.

*ADHD* Attention deficit hyperactivity disorder, *PTSD* Post-traumatic stress disorder

**Table 2:** Frequency distributions and Pearson’s chi-square values of potential mental health condition by gender

Potential mental health condition	Total sample (***N*** = 376)	Gender			
		Male (***N*** = 211)	Female (***N*** = 165)	χ^**2**^ (df = 1)	***P*** value
	***f*** (%)	***f*** (%)	***f*** (%)		
Mental health condition (any)	277 (73.7%)	147 (69.7%)	130 (78.8%) ↑	3.97*	0.046
Anxiety	115 (30.6%)	56 (26.5%)	59 (35.8%)	3.71	0.054
ADHD	11 (2.9%)	7 (3.3%)	4 (2.4%)	0.041	0.840^a^
Conduct Disorder	50 (13.3%)	31 (14.7%)	19 (11.5%)	0.81	0.368
Autism	11 (2.9%)	9 (4.3%)	2 (1.2%)	3.04	0.081^a^
Epilepsy	10 (2.7%)	8 (3.8%)	2 (1.2%)	1.49	0.223^a^
Motor Tics	9 (2.4%)	3 (1.4%)	6 (3.6%)	1.11	0.292^a^
Depression	136 (36.2%)	56 (26.5%)	80 (48.5%) ↑	19.31***	0.000
PTSD	100 (26.6%)	58 (27.5%)	42 (25.5%)	0.20	0.658
Social Phobia	26 (6.9%)	11 (5.2%)	15 (9.1%)	2.16	0.141
Learning Disability	4 (1.1%)	1 (0.5%)	3 (1.8%)	0.57	0.451^a^
Phobias	30 (8.0%)	16 (7.6%)	14 (8.5%)	0.10	0.749

*p ≤ 0.05; **p ≤ 0.01; ***p ≤ 0.001; ↑Higher scores; ^a^Continuity Correction applied

*ADHD* Attention-deficit-hyperactivity-disorder, *PTSD* Post-traumatic stress disorder

**Table 3:** Frequency distributions and Pearson’s chi-square values of psychosocial deprivation (PSD) by age group

PSD variables	Total sample (***N*** = 376)	Age group			
		4–9 Yrs. (***N*** = 208)	10–14 Yrs. (***N*** = 168)	χ^**2**^(df = 1)	***P*** value
	***f*** (%)	***f*** (%)	***f*** (%)		
PSD concern (any)	280 (74.5%)	149 (71.6%)	131 (78.0%)	1.97	0.161
War injury	28 (7.4%)	19 (9.1%)	9 (5.4%)	1.92	0.165
Child labour	45 (12.0%)	16 (7.7%)	29 (17.3%) ↑	8.08**	0.004
Loss of caregiver	36 (9.6%)	15 (7.2%)	21 (12.5%)	3.00	0.083
Neglect	51 (13.6%)	25 (12.0%)	26 (15.5%)	0.95	0.330
Domestic abuse	47 (12.5%)	28 (13.5%)	19 (11.3%)	0.39	0.530
Displacement	160 (42.6%)	82 (39.4%)	78 (46.4%)	1.87	0.172
Poverty	149 (39.6%)	68 (32.7%)	81 (48.2%) ↑	9.36**	0.002

*p ≤ 0.05; **p ≤ 0.01; ***p ≤ 0.001; ↑Higher scores

**Table 4:** Frequency distributions and Pearson’s chi-square values of psychosocial deprivation (PSD) variables by age group

PSD variables	Total sample (***N*** = 376)	Gender			
		Male (***N*** = 211)	Female (***N*** = 165)	χ^**2**^ (df = 1)	***P*** value
	***f*** (%)	***f*** (%)	***f*** (%)		
PSD concern (any)	280 (74.5%)	158 (74.9%)	122 (73.9%)	0.04	0.835
War injury	28 (7.4%)	19 (9.0%)	9 (5.5%)	1.69	0.193
Child labour	45 (12.0%)	40 (19.0%) ↑	5 (3.0%)	22.29***	0.000
Loss of caregiver	36 (9.6%)	14 (6.6%)	22 (13.3%) ↑	4.80*	0.028
Neglect	51 (13.6%)	24 (11.4%)	27 (16.4%)	1.97	0.161
Domestic abuse	47 (12.5%)	26 (12.3%)	21 (12.7%)	0.01	0.906
Displacement	160 (42.6%)	81 (38.4%)	79 (47.9%)	3.41	0.065
Poverty	149 (39.6%)	88 (41.7%)	61 (37.0%)	0.87	0.351

*p ≤ 0.05; **p ≤ 0.01; ***p ≤ 0.001; ↑ Higher scores

**Table 5:** Frequency distributions and Pearson’s chi-square values of social, behavioral and emotional (SBE) variables by age group

SBE variables	Total sample (***N*** = 376)	Age group			
		4–9 Yrs. (***N*** = 208)	10–14 Yrs. (***N*** = 168)	χ^**2**^(df = 1)	***P*** value
	***f*** (%)	***f*** (%)	***f*** (%)		
SBE concern (any)	244 (64.9%)	140 (67.3%)	104 (61.9%)	1.19	0.275
Low/abnormal socialisation	105 (27.9%)	69 (33.2%) ↑	36 (21.4%)	6.37*	0.012
Emotional issue	116 (30.9%)	66 (31.7%)	50 (29.8%)	0.17	0.681
Peer issue/being bullied	9 (2.4%)	6 (2.9%)	3 (1.8%)	0.13	0.724^a^
Peer issue/being aggressive	64 (17.0%)	28 (13.5%)	36 (21.4%) ↑	4.18*	0.041
Educational decline	56 (14.9%)	24 (11.5%)	32 (19.0%) ↑	4.13*	0.042

*p ≤ 0.05; **p ≤ 0.01; ***p ≤ 0.001; ↑ Higher scores; ^a^Continuity Correction applied

**Table 6:** Frequency distributions and Pearson’s chi-square values of social, behavioral and emotional (SBE) variables by gender

SBE variables	Total sample (***N*** = 376)	Gender			
		Male (***N*** = 211)	Female (***N*** = 165)	χ^**2**^ (df = 1)	***P*** value
	***f*** (%)	***f*** (%)	***f*** (%)		
SBE concern (any)	244 (64.9%)	132 (62.6%)	112 (67.9%)	1.15	0.284
Low/abnormal socialisation	105 (27.9%)	49 (23.2%)	56(33.9%) ↑	5.28*	0.022
Emotional issue	116 (30.9%)	61 (28.9%)	55 (33.3%)	0.85	0.357
Peer issue/being bullied	9 (2.4%)	5 (2.4%)	4 (2.4%)	0.00	1.000^a^
Peer issue/being aggressive	64 (17.0%)	45 (21.3%) ↑	19 (11.5%)	6.31*	0.012
Educational decline	56 (14.9%)	30 (14.2%)	26 (15.8%)	0.17	0.677

*p ≤ 0.05; **p ≤ 0.01; ***p ≤ 0.001; ↑ Higher scores; ^a^Continuity Correction applied

### Potential mental health conditions

The majority of the sample had at least one potential mental health problem (73.7%, *f* = 277), with nearly a third of the sample exhibiting symptoms of anxiety (30.6%, *f* = 115), depression (36.2%, *f* = 136) or PTSD (26.6%, *f* = 100) (see Fig. 1).

Testing for possible differences in the rate of potential mental health problems between the two age groups (M = 8.99, SD = 2.25 years of age) indicated that children from the ages of 4 to 9 years were more likely to have a potential mental health problem, *Χ*^2^(1, *N* = 376) = 1.26, *p* < .05, in particular symptoms of autism, *Χ*^2^(1, N = 376) = 4.42, *p* < .05, and PTSD *Χ*^2^(1, N = 376) = 4.15, *p* < .05, compared to those from the ages of 10 to 14 years.

When testing for possible gender differences in the rates of potential mental health problems, the girls were more likely to suffer than the boys from any potential mental health problem, *Χ*^2^(1, *N* = 376) = 3.97, *p* < .05, and show signs of depression, *Χ*^2^(1, *N* = 376) = 19.31, *p* < .001 (see Table 2). The remaining mental health problems did not vary between the girls and boys in this sample.

### Psychosocial deprivation concerns

Within the sample, 74.5% (*f* = 280) had at least one form of PSD present. Specifically, 42.6% (*f* = 160) were displaced and 39.6% (*f* = 149) suffered from abject poverty, as shown in Fig. 2 and Table 3.

Associations were found between age group and PSD variables as can be seen in Table 3. Adolescents (10–14 years) were more likely to be employed (17.3%) than the younger children (7.7%), with associations at *Χ*^2^(1, *N* = 376) = 8.078, *p* < .01. Additionally, nearly half (48.2%) of the adolescent age group suffered from abject poverty as compared to about one third (32.7%) in the younger age group, *Χ*^2^(1, *N* = 376) = 9.36, *p* < .001.

Gender differences were also present in the rates of child labour and loss of caregiver. Boys were significantly more likely to be in the child labour category *Χ*^2^(1, *N* = 376) = 22.29, *p* < .001, with 19% of the boys in the sample being employed, as compared to 3% of girls. Girls were more likely to be in the category loss of caregiver, *Χ*^2^(1, *N* = 376) = 4.80, *p* < .05.

### Social, Behavioural and emotional concerns

Of the entire sample, 64.9% (*f* = 244) exhibited some form of SBE concern. Nearly a third of the sample (27.9%, *f* = 105) displayed low or abnormal socialization behaviours, and 30.9% (*f* = 116) showed signs of emotional distress or concern. Frequency distributions and rates can be seen in Fig. 3 and Table 5.

The two age groups differed in the rates of one or more of the SBE variables as shown in Table 5. Younger age (4–9 years) was associated with increased likelihood of lower or abnormal socialization, *Χ*^2^(1, *N* = 376) = 6.37, *p* < .05. The older age group was more likely to show aggressive behaviour *Χ*^2^(1, *N* = 376) = 4.18, *p* < .05. The rate of aggression is nearly 8% higher when comparing aggression rates of the adolescent age group (21.4%) and the younger group (13.5%). The older age group was also more likely to show educational decline, compared to the younger age group, *Χ*^2^(1, *N* = 376) = 4.13, *p* < .05.

As shown in Table 6, boys were nearly twice as likely to exhibit aggressive behaviour towards their peers than girls (21.3% and 11.5% respectively). Male gender was associated with peer issues/being aggressive at *Χ*^2^(1, *N* = 376) = 6.31, *p* < .05. Additionally, female gender was associated with low or abnormal social behaviour *Χ*^2^(1, N = 376) = 5.28, *p* < .05 as compared to the male gender, with rates at 33.9 and 23.2% respectively.

### Regression models

The initial stepwise-covariate selection process revealed an association of six covariates with increased odds of the potential presence of mental health issues: these were social, behavioural and emotional (SBE) concerns, emotional issues, low/abnormal socialisation, war injury, displacement, and child labour. Of these, war injury and child labour were non-significant (Table 7).

**Table 7:** Logistic regression results for variables associated with mental health issues

Covariate	Odds ratio	***P*** value
(Intercept)	0.43 (95% CI: 0.19–0.92) *	0.037
Social, behavioural and emotional (SBE) concerns (any)	4.45 (95% CI: 1.68–12.7) **	0.004
Emotional issues	11.02 (95% CI: 2.76–74.49) **	0.003
Low/abnormal socialization	8.37 (95% CI: 2–57.71) **	0.009
War injury	32,786,952.72 (95% CI: 0 - NA)	0.989
Displacement	2.91 (95% CI: 1.21–7.48) *	0.02
Child labour	0.59 (95% CI: 0.21–1.64)	0.309

*p ≤ 0.05; **p ≤ 0.01; ***p ≤ 0.001

Emotional issues were most strongly associated with the presence of potential mental health issues, OR 11.02 (95% CI: 2.76–74.49), as well as low/abnormal socialisation, 8.37 (95% CI: 2–57.71). Significantly, but less strongly associated were SBE concerns, 4.45 (95% CI: 1.68–12.7) and displacement, 2.91 (95% CI: 1.21–7.48).

Stepwise-covariate selection of variables associated with case improvement only identified two covariates, child labour and educational decline. Of these, only child labour was significantly associated with a reduction in the odds of case improvement, 0.24 (95% CI: 0.07–0.92) as seen in Table 8.

**Table 8:** Logistic regression results for variables associated with case improvement

Covariate	Odds ratio	***P*** value
(Intercept)	6.72 (95% CI: 4.17–11.5) ***	0
Child labour	0.24 (95% CI: 0.07–0.92) *	0.03
Educational decline	0.52 (95% CI: 0.22–1.33)	0.16

*p ≤ 0.05; **p ≤ 0.01; ***p ≤ 0.001

## Discussion

### Contribution to program design and support

Firstly, the research aimed to empirically document and analyse prevalence rates of 23 different mental health and psychosocial support (MHPSS) and protection sector variables in a sample of 376 school children in the Northwest of Syria. Secondly, we tested for associations between the demographic variables of age group and gender with the presence of potential mental health conditions, psychosocial deprivation (PSD) variables, and social, behavioural and emotional (SBE) variables in the sample. Thirdly, we examined which of the demographic (age group, gender) and 23 protection sector variables affected potential mental health and protection/MHPSS intervention outcomes. Using case progress outcomes for a single MHPSS protection project in Syria, we found that numerous PSD and SBE covariates significantly and substantially increased the odds of having a potential mental health concern, most notably indications of emotional issues and low/ abnormal socialisation. After the conclusion of planned interventions, the project noted case improvement for 63.6% (*f* = 239) of the children in the sample, and was least effective for children who are working. Overall, there was a case decline or regression in 14.4% (*f* = 54) of the cases, and the data was incomplete for 22.1% (*f* = 83). Case decline and incompleteness was associated with child labour. These associations serve to showcase the unique needs and trends within a given sample, with this methodolthese early stages to minimiseogy potentially supporting project tailoring and the refinement of MHPSS activities to better serve beneficiaries and improve intervention success rates. Such methods may effectively maximise the use of limited resources, and may improve the quality of the services provided, while potentially minimizing risks posed to the sample in the cases where case decline is noted. MHPSS project implementation often requires some degree of trial-and-error when initiating projects in a new cultural context, potentially benefitting from such data-driven analysis in these early stages to minimise negative impacts down the line.

Field work is typically guided by best practice standards, such as those presented in the INEE Mental Health Standards and Indicators, the Sphere Handbook, the CPMS guide, the Child Protection in Emergencies Handbook by the GPC, and various tools by the Inter-Agency Standing Committee (IASC) such as the IASC MHPSS Guidelines for Emergency Settings and the IASC Monitoring and Evaluation (M&E) Framework for MHPSS in Emergency Settings; which combines efforts of 27 well-established agencies including the United Nations (UN) and similar actors [18, 37–39]. The use of more specialized data analysis can aide and support the use of these tools in the field, where the wide variety of recommended actions can be narrowed down to better suit the unique needs of the beneficiaries and potentially improve project outcomes. In this particular sample for example, it was identified that employed children were less likely to see a successful MHPSS intervention. With this knowledge, creative solutions, activities, or links to additional specialized support services could be utilized to better support these children in the sample. Our findings around employment also support the known intersectionality issues that revolve around child employment and gender, and the related socioeconomic factors that are often difficult to address in a standalone MHPSS project. The present research confirms most of what established MHPSS guidelines advise, including the need for streamlined interventions across sectors to address the variety of factors that contribute to increased risk of PSD and poor mental health in children in humanitarian contexts [1, 18, 19, 21, 24, 33].

MHPSS and protection interventions can also be differentiated from others in that they often depend on projects from other sectors, and require a high level of coordination, organic tailoring, and sensitivity to each unique community’s needs and cultures. Looking at the activities implemented in this current sample for example, MHPSS methodologies often relied on other projects to provide the most basic needs of shelter, security, livelihood support, education, and healthcare coordination where needed. Such methodologies in emergency settings, such as in the Northwest of Syria at present, often require such an approach that incorporates sustainable multi-sector project integration so as to provide the most essential needs of a child, and to avoid any unintended negative consequences such as social stigma due to specialized mental health treatments [18, 21]. That being said, specialized mental healthcare is often the most effective approach at addressing the significantly increased mental health issues associated with conditions of adversity [18]. Such specialized mental health services remain highly underutilized and often receive significantly less support in FCAS, including in the Northwest of Syria [1, 44]. While cultural perceptions and social stigma stand as one of the many barriers for access to such services, the severity of this conflict and the associated mental health concerns that ensue may present an opportunity for the development of such programs. MHPSS projects that include awareness activities can support community level advocacy for specialized mental health services to be more readily available and accepted in Syria [25, 44, 45].

With such cultural sensitivities to the subjects around protection and MHPSS still present in Syria however, a number of factors have to be considered when planning for protection projects, such as planning for effective and socially appropriate entry points into the community. These provide opportunities for protection projects to be more readily accepted, often integrated through other projects and already established common spaces. This ultimately allows for increased community awareness and the implementation of more tailored protection and MHPSS activities such as gender-based violence (GBV) support, guided storytelling, service provider mapping, structured activities through schools, community centres, and hospitals, community strengthening activities, and in some cases specialized treatment [13, 18, 21, 37, 38]. Our findings highlighted both the gender and age associations with the presence of potential mental health concerns, touching on sensitive topics in Syria around female and children’s mental health. Identifying such demographic patterns within a sample may help identify higher risk beneficiaries, appropriate project entry points, guide awareness campaigns and activities, and initiate additional efforts to support and understand the underlying causes of increased depression in girls, and PTSD in the younger children’s age group for example.

### Contribution to psychiatric research

Presently, civilians including children from this sample are subject to targeted shelling and are at an especially high risk of being exposed to violence [46]. Research from within the country is extremely limited, and beyond benefits to project tailoring; such project-level analysis can offer valuable insights into the state of children’s mental health during the Syrian conflict. Save the Children’s report on the mental health of Syrian children (2017) found that over 84% of adults and nearly all children expressed fears of shelling and bombing as their primary concern during the conflict [14]. The parents of these children have reported higher rates of aggression, and 71% said their children suffer from involuntary urination – a noted symptom of anxiety and toxic stress [15]. Similar symptoms and behaviours; including developed speech impediments and phobias were noted in the casefiles of our student sample. Older students and male students in the sample were also associated with aggressive behaviour, with aggression concerns observed in 17% of all cases. Save the Children’s report, among others, found that the majority of children who had seen violence, lost a parent or been exposed to aerial bombardment are reported to have increased likelihood of phobias and anxiety [14, 29, 40].

The majority of Syrian children have very little access to mental health professionals, and often come from homes with family members that suffer similar or worse mental and physical health conditions [14, 47–50]. By 2019, nearly half of the sample (42.6%) was already displaced, and well over a third was suffering from abject poverty (39.6%). Our findings indicated that displacement was associated with a the presence of potential mental health issues, which supports field research and observations around ‘migration stressors’, and the impacts of forced migration [7, 15]. Lack of support from trusted adults and deterioration of communities is highly linked to increased prevalence of toxic stress, and may reduce the likelihood of having protection concerns resolved [15, 27, 38, 49, 50]. The current research findings indicate that a variety of demographic features, including gender and young age, can place certain children at higher risk for depression, anxiety, and general presence of PSD and SBE concerns. In our sample, 73% of children suffered from a potential mental health concern; with 30.6% indicating symptoms of anxiety, 36.2% depression, and 26.6% indicating signs of PTSD. Girls and younger children were also found to be at an increased risk of having potential mental health concerns, with girls being higher risk for indications of depression and younger children being higher risk for indications of PTSD. These findings echo other psychiatric studies that estimate approximately 22.1% of those living in FCAS will suffer from PTSD and depression, and research indicating that girls are more likely to exhibit symptoms of depression and anxiety [14, 23, 32, 45].

In recent years the protection sector has seen significant improvements, with MHPSS projects like this one exhibiting increasing rates of success, despite often taking a secondary priority to other disaster response interventions [34]. Funding that goes to mental health programs and specialized mental healthcare in FCAS remains extremely low, being only 1% of the global emergency response budget and meeting only 35% of projected funding needs in 2015 [6]. Research on mental health during conflict and adversity is often extremely difficult to implement, despite the fact this may effectively support larger mental health and protection advocacy efforts, as well as practices in the field [21, 27, 35, 44]. With such populations being consistently on the move, susceptible to social stigma, or unwilling to relive their experiences; in depth or long-term data can be difficult to obtain and it’s especially difficult to predict how communities will react to information gathering in times of extreme adversity [18, 21]. As such, organically collected data through such protection and MHPSS projects can offer a more cost-effective solution to these gaps in research, providing high volumes of data without being intrusive. In particular, additional research on the factors that predict resilience or general mental health outcomes during conflict could inform psychiatric practice in Syria and other FCAS. The most prevalent protection concerns in the current sample were related to mental health issues and PSD concerns, and additional research that identifies relationships among the two may help inform psychiatric practice, project planning, and larger advocacy efforts.

### Study limitations

There are a number of limitations present in the study which should be considered. Firstly, the creation of variables and screening of the data (particularly the mental health data) was done retrospectively, using screening tools on qualitative data. However, since the staff trainings, notes, and senior level case management were conducted by trained mental health staff who are experienced in standardized protection data gathering in this context, we found the data to be sufficient for the aims of this research. The rates of potential mental health conditions reflect this, in that the figures echo much of what other psychiatric research has already established [32, 45]. Overall, the data and results reflected much of the expected outcomes and mirrored observed patterns (i.e. associations between gender and employment, gender and mental health, age group and employment, etc.) [1, 16, 32]. Similarly, limitations are present in measuring case outcomes (improvement, decline or incompleteness), which was indicated subjectively by the case managers. These were indicated for each child with noted justifications, and there is a risk of bias present in such indications. As noted, staff are trained in MHPSS case management and their observations on case outcomes were deemed appropriate for the purposes of this research.

Cases that included child labour was also limited in some aspects, with some cases only noting MHPSS intervention outcomes, without as much information on mental health, SBE and PSD. From the data, correlation with child labour and other mental health, PSD and SBE issues were negative, reflecting the limited data available. The case managers are often unable to collect in-depth details on children who are employed due to the absence of the child from school.

Data on children who are displaced may have yielded results that are not truly representative due to the nature of the sample. While a variety of studies confirm that children who are IDP’s have significantly higher mental health, PSD and SBE concerns, the delineation is difficult to indicate in this particular sample, as it is limited to a sample of children who are already of concern and not a random sample that is reflective of normal population distributions in Idleb.

More specific physical health variables (i.e. chronic illnesses) were also excluded from this research and would warrant further testing to assess for comorbidity and overlap between physical health and the discussed protection and MHPSS variables. This project was implemented and managed by experienced educators, MHPSS and protection case workers and not healthcare providers. Additionally, children with physical health concerns receive healthcare elsewhere, and as such it would be difficult to determine the effectiveness of MHPSS interventions on these children.

Finally, a large number of statistical tests were conducted, raising the possibility of Type I error inflations. Most of our findings, however, echoed other research on associations between mental health, PSD and SBE, and therefore likely to represent true effects (in some cases with small effect sizes) in this sample. Additionally, chi-squared independence tests was were done separately and each computed on a 2 × 2 table with *p*-value significance differentiated at three different levels (*p* ≤ 0.05, *p* ≤ 0.01 and *p* ≤ 0.001) to account for potential Type I error inflations. Continuity corrections were applied where variable frequencies (*f*) were less than 5.

## Conclusion

The results of this research essentially showcase the improvement of the majority of the children in the sample after the implementation of MHPSS activities, and highlights areas of potential tailoring based on gender, age group, or specific protection factors. This retrospective analysis can support the development of new methodologies to be used on projects in real-time, to analyse the unique beneficiary case details and outcomes for empirical associations and patterns. Ultimately, this can help guide MHPSS activity tailoring throughout the project lifecycle – which can potentially improve project outcomes, and flag risks that may not have been identified otherwise. MHPSS intervention outcomes require more in depth understanding of the relationships between social factors, and the mental and physical wellbeing of beneficiaries than other sectors, and as such warrant more in depth understanding of the beneficiaries and their individual needs. Beyond this, research from inside Syria is extremely limited – and populations in neighbouring countries face significantly different contexts and concerns than children and populations still inside Syria. Project analysis can serve to fill this gap in knowledge, through already established access that can provide of organic and non-intrusive data.

This research presents and supports findings on the links between various demographic, protection and MHPSS variable concerns in the Northwest of Syria, and can serve as an example of empirical project research that may lead to real-life solutions and advocacy for practitioners in FCAS. Our results reflect the highly complex nature of MHPSS variables, needs and outcomes in Syria, and highlight the evidenced case improvements as seen by PSS projects on the ground. This ultimately calls for the need to implement far-reaching and intersectoral approaches in children’s protection projects, and for the integration of protection standards across various projects and sectors. While allargescale research and more specialized support is gravely needed in Syria, standard practice in the field could benefit from such in depth project analysis methodologies, to further advocate for and inform the sector overall. The importance of prioritizing such protection projects in FCAS can have significantly positive effects on the future outlook of conflict ridden communities, both in Syria and elsewhere.

## Data Availability

The data used and analysed during the current study are available from the corresponding author on reasonable request; access is highly limited due to the ongoing conflict in Syria, and the sensitivity of information of those represented in the sample.

## Abbreviations

ADHD: Attention Deficit Hyperactivity Disorder
CFS: Child-Friendly-Space
CSI-4: Child Symptom’s Inventory IV
FCAS: Fragile and Conflict Affected Setting
GBV: Gender-Based Violence
GPC: Global Protection Cluster
IASC: Inter-Agency Standing Committee
IDP: Internally Displaced Person
M&E: Monitoring and Evaluation
MHPSS: Mental Health and Psychosocial Support
NGO: Non-Governmental Organization
PSD: Psychosocial Deprivation
PSS: Psychosocial Support
PTSD: Post-Traumatic Stress Disorder
SBE: Social, Behavioural and Emotional
UN: United Nations
WHO: World Health Organization
YI-4R: Youth-Inventory IV

## Footnotes

1 Including: Attention Deficit Hyperactivity Disorder (ADHD), Oppositional Defiant Disorder (ODD), Conduct Disorder, Generalized Anxiety Disorder, Social Phobia, Separation Anxiety Disorder, Obsessive-Compulsive Disorder (OCD), Specific Phobia, Major Depressive Disorder, Dysthymic Disorder, Schizophrenia, Pervasive Developmental Disorder, Asperger’s Disorder, Motor Tics, and Vocal Tics

2 Including: ADHD, ODD, Conduct Disorder, Generalized Anxiety Disorder, Social Phobia, Separation Anxiety Disorder, OCD, Specific Phobia, Panic Attacks, Major Depressive Disorder, Dysthymic Disorder, Bipolar Disorder, Schizophrenia, Motor Tics, Vocal Tics, Schizoid Personality Disorder, Somatization Disorder, Anorexia Nervosa, Bulimia, and Drug Use
